# Impact of high-intensity interval training vs. moderate-intensity continuous training combined with strength training on physical and metabolic outcomes in post-bariatric surgery patients with sarcopenic obesity

**DOI:** 10.1186/s12891-026-09722-z

**Published:** 2026-04-07

**Authors:** Mastaneh Rajabian Tabesh, Alireza Khalaj, Maryam Mousavi, Maryam Abolhasani, Fatemeh Shabkhiz, Rahman Soori

**Affiliations:** 1 Department of Exercise Physiology, Faculty of Sport Sciences and Health, Alborz Campus, University of Tehran, Tehran, Iran; 2 Obesity Treatment Center, Department of Surgery, Shahed University, Tehran, Iran; 3 Department of Clinical Nutrition and Dietetics, National Nutrition and Food Technology Research Institute, Faculty of Nutrition and Food Technology, Shahid Beheshti University of Medical Sciences, Tehran, Iran; 4 Cardiac Primary Prevention Research Center, Cardiovascular Diseases Research Centre, Tehran University of Medical Sciences, Tehran, Iran; 5 Department of Exercise Physiology, Faculty of Sport Sciences & Health, University of Tehran, Tehran, Iran

**Keywords:** Bariatric/metabolic surgery, High-intensity interval training, Moderate-intensity continuous training, Sarcopenic obesity

## Abstract

**Background:**

Sarcopenic obesity (SO) is a pathological condition characterized by a decline in skeletal muscle mass accompanied by increased fat accumulation. This condition is associated with a higher risk of physical limitations, cardiovascular diseases, and weight loss failure following bariatric surgery (BS). To identify more effective management strategies for SO after BS, this study aimed to compare the effects of high-intensity interval training (HIIT) and moderate-intensity continuous training (MICT) on body composition parameters, physical performance, and cardiometabolic indicators in patients with SO post-BS.

**Methods:**

In this study, 80 females with body mass index 40 ≤ BMI ≤ 50 kg/m2 who were candidates for undergoing BS and diagnosed with SO were recruited. Participants were divided into two groups: HIIT (*n* = 40) and MICT (*n* = 40), each further subdivided into sleeve (*n* = 20) and bypass (*n* = 20). Both interventions were followed over an 8-week period after BS, starting from four to 12 weeks post-surgery. Body composition (Weight, Fat Mass, Skeletal Muscle Mass, and Fat Free Mass), physical performance (aerobic capacity, physical ability, and muscle strength), and cardiometabolic indicators (glycemic indicators, lipid profile, and inflammatory factors) were measured pre and post intervention using body impedance analyzer, standardized functional tests, and blood samples, respectively. A 24-hour food diary was also obtained by conducting an in-person interview for food intake assessment.

**Results:**

A total of 80 participants completed the study. Age was a significant covariate (*p* = .007). After adjusting for age, HIIT significantly reduced weight, BMI, calorie intake, and carbohydrate intake compared to MICT, with the HIIT-bypass group showing significant improvements in HbA1c, insulin, and FBS (mean reduction of 7.71%, 24.98%, and 6.34%, respectively; *p* < .05). Additionally, HIIT-bypass reduced hs-CRP (mean reduction of 23.81%) and increased HDL (mean increase of 8.88%) (*p* < .001). In terms of physical performance, while HIIT in both the sleeve and bypass groups significantly improved Sit-to-Stand performance (+ 26.97% for sleeve and + 27.47% for bypass, *p* < .05), the HIIT-sleeve group showed the greatest improvements in walking speed and balance test (mean increase of 10.33% and 14.59%, respectively, *p* < .001), whereas HIIT-bypass exhibited the most significant improvement in Timed Up-and-Go (+ 12.21%, *p* < .001). These findings highlight the superior effectiveness of HIIT over MICT (dependent on the type of surgery) in improving both metabolic and some physical outcomes.

**Conclusion:**

Significant differences were observed in cardiometabolic and physical performance parameters between the two training protocols. HIIT resulted in significant improvements in aerobic capacity, physical ability, glycemic indicators, hs-CRP, HDL, weight, BMI, and food intakes compared to MICT. These findings may position HIIT as a promising intervention for optimizing metabolic and physical outcomes in post-bariatric patients with sarcopenic obesity. However, no significant changes were observed in muscle strength or skeletal muscle mass. These findings position HIIT as a promising intervention for optimizing certain metabolic and physical outcomes in this population, though further blinded, randomized controlled trials are warranted to confirm these results.

**Authorized ethics committees Number:**

IR.TUMS.THC.REC.1401.052.

**Trial registration:**

Current research is a prospective quasi-experimental study, and clinical trial number is not applicable.

## Background

Bariatric surgery (BS) is the most effective and promising treatment for morbid obesity [1, 2]. However, due to various factors, this surgery can also result in unsuccessful weight loss outcomes, often marked by a disproportionate reduction in fat-free mass (FFM) while retaining a higher percentage of fat mass (FM) [3–5]. Sarcopenic obesity, characterized by a decline in skeletal muscle mass (SMM) and function alongside increased fat accumulation, has been identified as a key predictor of negative outcomes following BS [6]. This condition not only diminishes quality of life but is also prevalent among BS candidates [7]. Notably, since BS leads to reductions in both FM and skeletal muscle and bone mass [8, 9], these changes can exacerbate muscle atrophy and impair physical performance in patients with sarcopenic obesity. This condition makes them more susceptible to unfavorable weight loss outcomes compared to other BS candidates, emphasizing the importance of addressing this disorder post-surgery. Therefore, identifying appropriate treatments alongside BS is crucial for achieving optimal weight reduction and improving obesity-related comorbidities, such as cardiovascular health, in patients with sarcopenic obesity.

The strategies for sarcopenic obesity management are focusing on improvements in body composition measures and physical performance as well as reduction in cardiometabolic indicators. However, so far, no definitive drug method has been approved for the management of sarcopenic obesity, and the most common treatment methods are based on lifestyle interventions, including physical activity and nutritional interventions [10]. Nutrition primarily affects the condition through insufficient protein intake, an imbalance between energy intake and expenditure, excessive fat accumulation, nutritional deficiencies, and suboptimal bone mineral density [11]. Additionally, many studies have indicated that physical activity plays an important role in improving sarcopenia in different age groups [11, 12].

Modifying nutritional intake after BS can be highly challenging, making physical activity a critical focus for managing sarcopenic obesity. For individuals who have undergone BS and experienced suboptimal weight loss, exercise serves as an effective complementary therapy. A previous review highlighted the positive effects of exercise on anthropometric measures, cardiovascular risk factors, and physical fitness in post-BS patients, positioning physical activity interventions as a promising strategy following BS [13]. According to the American College of Sports Medicine (ACSM) recommendations, majority of individuals without complications, one month after surgery can engage in moderate- or vigorous-intensity exercise throughout the week [14, 15]. However, the main question, which is remained unanswered, is that what the most effective exercise is in the shortest time. Considering the lack of time, facilities, and costs, this question can show the importance of the type of exercise for people who are seeking weight loss.

Evidence supports the positive effects of two well-known types of exercise, high-intensity interval training (HIIT) and moderate-intensity continuous training (MICT), in improving parameters related to sarcopenic obesity, such as body composition and cardiometabolic indices [16–18]. MICT is an aerobic exercise model that is considered a successful intervention for reducing FM and metabolic disorders in obese adults. On the other hand, HIIT is a short-duration endurance exercise often prescribed to patients with and without cardiovascular diseases due to its positive impact on the cardiovascular system in a shorter exercise session [18, 19]. Although MICT may provide slower results, the impact of HIIT on body mass and composition is controversial [18, 19]. Generally, the results for both training protocols (HIIT and MICT) on body composition, cardiovascular parameters, and physical performance were inconsistent. Therefore, further studies are required to confirm the precise effects of HIIT and MICT on parameters associated with sarcopenic obesity.

Few studies have investigated the effects of HIIT and MICT in BS patients, and those that exist are insufficient for guiding clinical practice which mainly focused on general obesity populations. Notably, two studies specifically compared HIIT and MICT protocols in sarcopenic obesity, showing different results in determining the superior type of training for this condition [17, 20]. The first study demonstrated that MICT was more effective than HIIT in reducing the expression of muscle atrophy marker genes in obese mice with muscle atrophy [17]. In contrast, the second study found that HIIT had a significantly greater impact on improving myocardial dysfunction in obese sarcopenic rats compared to MICT [20]. Although evidence comparing HIIT and MICT in patients with sarcopenic obesity is lacking, a study in older adults with obesity, which might resemble sarcopenic obesity, reported greater improvements in functional capacity, lean mass, and skeletal muscle markers with HIIT than MICT [19].

To the best of our knowledge, no studies have directly compared HIIT and MICT in post-bariatric patients with sarcopenic obesity. However, this condition significantly increases the susceptibility of BS candidates to unfavorable weight loss outcomes compared to other BS candidates, underscoring the importance of addressing this disorder post-surgery. The early postoperative period, particularly the first six months after BS, represents a critical window for intervention, as this period significantly shapes long-term outcomes. Targeted physical interventions during this time can greatly influence recovery and the management of comorbidities [21].

According to inconsistent evidence regarding the effects of HIIT and MICT in animal models and older adults with obesity, combined with a lack of studies comparing these training modalities in the BS population, there is a critical gap in understanding their relative efficacy for improving cardiometabolic health and physical performance. This study addresses these gaps by evaluating the comparative effects of HIIT and MICT on various body composition parameters, aerobic capacity, physical ability, muscle strength, and cardiometabolic indicators in patients with a history of sarcopenic obesity eight weeks post-BS. This could help health practitioners provide better solutions for managing sarcopenic obesity, thereby improving outcomes post-surgery.

## Materials and methods

### Study design

This is a prospective quasi-experimental study, which approved by the authorized ethics committees (No: IR.TUMS.THC.REC.1401.052), and informed consent was obtained from all participants. Participants were informed about the study’s purpose, assessments and associated risks. The current study investigated the effects of 8 weeks of HIIT and MICT, combined with strength training, on various health parameters in participants who were scheduled for BS and diagnosed with sarcopenic obesity before the surgery by a sports medicine specialist. The exercise intervention method involves dividing the patients into two groups: HIIT and MICT, with both groups receiving the same strength exercise protocol. Additionally, type of surgery procedures was matched between the two groups. It should be noted that this is not a blind intervention.

### Participants

The participants referred to Hakim Medical Center in Tehran, Iran, were included in the study according to the following inclusion and exclusion criteria. The inclusion criteria for the study were (1) females (2) aged 20 to 50 (3) who were candidates for gastric bariatric surgery and (4) had a body mass index (BMI) between 40 and 50 kg/m2 (5), along with sarcopenic obesity. Exclusion criteria included (1) pregnancy during the study (2), musculoskeletal problems or (3) neurological disorders that could impact functional tests (4), the presence of pacemakers or other electrical devices that could interfere with body composition analysis (50, the use of weight-affecting medications like appetite suppressants (e.g. SLIMQUICK, SLIM LAST, etc.) (6), taking non-prescribed supplements (7), unwillingness to cooperate (8), uncontrolled diabetes (9), severe cardiovascular diseases (10), skeletal muscle limitations (11), severe neurological disorders (12), uncontrolled hypertension (13), severe chronic obstructive pulmonary diseases (14), simultaneous surgeries, or (15) a history of prior weight loss surgeries.

Eighty females (*n* = 80) completed the intervention and matched for their two types of surgery procedures (sleeve and bypass). Among these participants 40 received HIIT and 40 received MICT. Furthermore, within each group of HIIT and MICT, they are further divided into two subgroups: sleeve (HIIT-sleeve = 20 and MICT-sleeve = 20) and bypass (HIIT-bypass = 20 and MICT-bypass = 20). Participants were considered to have completed the intervention if they attended at least 70% of the training sessions (minimum: 17 out of 24 sessions) and completed both the pre- and post-intervention evaluations.

### Diagnosis of sarcopenic obesity

Sarcopenic obesity does not have a standalone ICD code but can be classified using the codes for sarcopenia (M62.84) and obesity (E66). Our sports medicine specialist used the following formula and handgrip test to diagnose sarcopenic obesity in individuals, and females diagnosed with sarcopenic obesity were included in the study. Jung Hee Kim et al. utilized the formula below to estimate the Appendicular Skeletal Muscle mass (ASM) value for bioimpedance [22]: ASM (kg) = [(Ht (2)/R × .104) + (age × -0.050) + (sex × 2.954) + (weight × 0.055)] + 5.663. In this formula, Ht represents the height in centimeters, R is the BIA resistance at 250Ω, and sex is denoted by 1 for men and 0 for females, along with age in years. We used the Asian Working Group for Sarcopenia (AWGS) 2019 criteria for threshold cutoffs for ASM, which is < 18 kg for women [23]. It should be noted that this method, which uses BIA, is a recognized approach for field-based studies, though DXA is the gold standard. Furthermore, handgrip test was also used for sarcopenic obesity diagnosis. The instrument used to measure hand strength was the Jamar dynamometer, and the maximum pressure applied to the dynamometer handle recorded as hand strength [24]. The average of three attempts was noted as the strength of each hand.

### Exercise intervention and randomization

ACSM recommends walking with a gradual increase in speed and duration, up to 150 min per week, during the first 4 weeks after surgery. Thus, according to these guidelines for physical activity intervention after BS, the 8-week intervention in the current study started during the second 4-week period post-BS [14, 15].

**Fig. 1:**
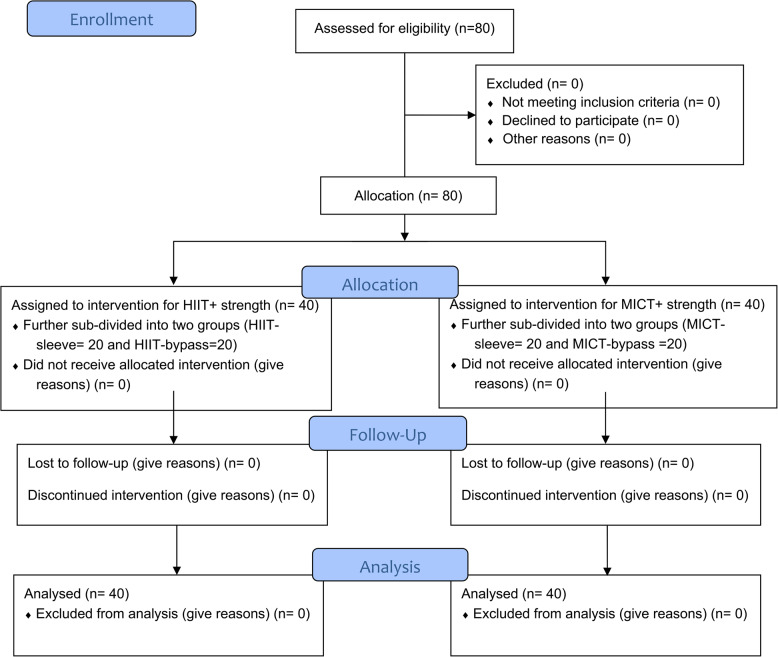
Study population and intervention flowchart, showing recruitment, allocation, follow-up, and dropouts/missing (if any). n= population number, HIIT=High Intensity Interval Training, MICT= moderate intensity continuous training

Participants were initially allocated to HIIT or MICT groups based on convenience. Subsequently, block randomization (block size = 4) was applied within these groups to ensure balanced allocation to sleeve and bypass surgery subgroups (Fig. 1). The random list was prepared using black stratified randomization software version 6.0.

Consent was obtained from both groups before entering the study that they will be ready for 8 weeks of exercise and three times a week. Participants performed their HIIT and MICT based on the training protocols described in Table 1, which were prescribed by a sports medicine specialist. To avoid impact and injuries during the first session, the specialist carefully instructed and trained the participants.

**Table 1:** HIIT, MICT, and strength protocols

HIIT Protocol		
Frequency	3 times per week	
Intensity	Warm up at 65%–70% of HR Reserve, according to the individuals’ tolerance level Exercise at 85%–90% of HR Reserve, according to the individuals’ tolerance level Active recovery at 65%–75% of HR Reserve, according to the individuals’ tolerance level Cooldown	
Type	treadmill walking	
Time	Exercise 4 intervals of 4 min 3 min active recovery in interval in between 3-min cooldown 30 min/session	
MICT Protocol		
Frequency	3 times per week	
Intensity	65%–75% of HR Reserve, according to the individuals’ tolerance level	
Type	Treadmill walking	
Time	40 min/session	
Strength protocol		
Repetitions		8 to 12 repetitions per movement, 1 to 3 sets for each limb
Frequency		3 session per week
Type		Using elastic band for Shoulder and for hip

*HIIT *High-Intensity Interval Training, *MICT *Moderate-Intensity Continuous Training, *min *minutes

During the exercise training session, participants were instructed to use the Rating of Perceived Exertion (RPE) scale to gauge their effort, as not all had access to reliable digital heart rate monitors at home. The RPE scale measures perceived fatigue in relation to heart rate. An RPE of 12–16 corresponds to moderate intensity, while 17–18 indicates high intensity. Participants recorded their RPE in a logbook. For progression, when participants reported an RPE of 12–16, they were advised to increase exercise intensity to reach an RPE of 17–18, according to the individuals’ tolerance level. Heart rate was monitored during exercise under the supervision of a supervisor using the Karvonen formula:

- HR rest = Heart rate after 3 min of sitting rest.
- HRmax = 220 – age.
- HR reserve= HRmax- HR rest.

In the above formula, the value of 65%–75% of HR Reserve used for the percentage of MICT, and 85%–90% of HR Reserve considered for the HIIT. Warm-up was done on a treadmill for 3 min at the speed mentioned in the Table 1. Cool-down was done until the heart rate reached a normal level. For progression, it started with minimum intensity (minimum and maximum intensity are written in the protocol in the Table 1) and was increased to maximum intensity according to individual tolerance.

Strength training in both groups was performed using elastic bands. Training with elastic bands was taught to the patients under direct supervision (3 sessions per week, 8 to 12 repetitions per movement, and 1 to 3 sets for each limb). The strength movements with the elastic band for the shoulder included extension, flexion, abduction, and adduction, while for the hip, they included extension, flexion, abduction, and thigh adduction. If patients reached the level of 3 sets of 12 repetition in strength training and the exercise with the bands became easy, a band of higher resistance was provided to them free of charge. All of these exercises were taught to the participants alongside their HIIT and MICT protocols (Table 1). Prescribed according to strength level (low, moderate, and high), color, and the average measurement of the maximum one-repetition maximum, using either red, green, or blue bands (3 days a week for 20 to 30 min).

Patients were given a sports training booklet during the training session by a sports medicine specialist, and after teaching their intervention protocol, they performed the home-based exercise intervention and filled out the logbook for the intervention and their adherence level and possible harm. The following complications were monitored during and after the exercise session: (1) Typical or atypical chest pain (2) Inappropriate palpitations (3) Fatigue or symptoms of overtraining (4) Joint pain or injury (5) Back and spine issues (6) Vertigo, headache, dizziness, or lightheadedness (7) Persistent shortness of breath (8) Hypo- or hypertensive episodes (9) Fainting. Because some patients lived far from the clinic and we did not have access to all of them for follow-up, they did not receive in-person exercise supervision and were contacted by phone instead. The participants completed their logbooks every week and submitted them.

### Outcomes measured

The strategies for sarcopenic obesity management are focusing on improvements in body composition measures and physical performance as well as reduction in cardiometabolic indicators. All the measurements including, nutritional intake, body composition factors, cardiometabolic indicators (biomedical measurements), and physical performance tests were taken before and after the intervention. Additionally, to evaluate physical activity status, the Global Physical Activity Questionnaire (GPAQ) [25] was used, which was completed by the participants before the intervention to ensure homogeneity among the participants. The validity and reliability of this tool have been reviewed and approved by the World Health Organization in 9 member countries, including Iran [26, 27].

#### Nutritional intake

In order to evaluate the food intake, a 24-hour food diary was obtained by conducting an in-person interview by a trained dietitian [28]. This tool compared against weighed food records, with a validity correlation of *r* = .60–0.85. After completing the 24-hour oral recall, then, the information obtained through this method, was analyzed using N4 nutritional software, and the daily intake of each person determined in terms of total energy, protein, carbohydrates, and fat. After the food intake assessment, all patients were giving a standard diet (600–800 kcal for the first 4 weeks after surgery, 800–1000 kcal for the second 4 weeks and 1000–1200 kcal with a high protein formula for the duration of the plan) by a nutritionist. Finally, the assessments were performed before and after the 8 weeks of intervention.

#### Body composition factors

The height of the participants was measured using a standard height meter. Weight and other body composition measurements were conducted by a trained expert with the Inbody 370 Biospace America, Inc. body impedance analyzer (BIA) device. BIA is widely validated against dual-energy X-ray absorptiometry (DXA) with reliability coefficients (intraclass correlation coefficient (ICC) ranging from 0.85 to 0.98 in obese populations. Prior to the BIA analysis, participants were given the following necessary instructions: (1) refrain from eating or drinking for four hours before the analysis on the day of the visit. (2) Avoid exercising 12 h before the test. (3) Do not take diuretics (diuretic drugs) or consume alcohol before the test. (4) Limit the intake of diuretic foods such as caffeine and chocolate before the test, and empty the bladder half an hour before the analysis. Finally, the assessments were performed before and after the 8 weeks of intervention.

#### Biochemical measurements

Blood samples were collected after a 12-h fast and without strenuous exercise for the previous 24 h. Fasting glucose (FBS), triglycerides (TG), cholesterol, and high-density lipoprotein cholesterol (HDL-c) were measured using an automated enzymatic method (Autoanalyzer; Technicon, Tarrytown, NY, USA). Low-density lipoprotein cholesterol (LDL-c) was calculated using the Friedwald formula. Insulin was determined by chemiluminescence immunoassay. Glycated hemoglobin (HbA1c) was determined by high-performance liquid chromatography and high-sensitivity quantitative C-reactive protein (hs-CRP) was quantified by turbidimetry. Finally, ferritin was measured using immunoturbidimetry test. These assessment methods are widely used in clinical research with high-sensitivity. Additionally, the assessments were performed before and after the 8 weeks of intervention.

#### Physical performance

All physical performance tests including aerobic capacity, physical ability and muscle strength were assessed by a sports medicine specialist before and after the 8 weeks of intervention.

##### Aerobic capacity

We assessed aerobic capacity using the 6-Minute Walking Test (6MWT) and the Walking Speed test [29]. ICC > 0.90 in bariatric patients, validated against cardiopulmonary exercise testing. We administered the 6MWT by asking participants to walk as far as they could in 6 min along a 30-meter-long path that had already been set up in the clinic. The distance walked was recorded. Lastly, we measured walking speed using a stopwatch for each participant.

##### Physical ability

We used the Up-and-Go, Sit-to-Stand, and Balance tests for physical ability assessment. The Sit-to-Stand test was performed using a chair without handles, approximately 44.5 cm high and 38 cm deep. The chair was positioned against the wall to minimize the risk of falling. Participants were instructed to stand and sit completely without using their hands for assistance. They were allowed to rest if needed. Participants repeated the task until 30 s had completed, and the number of times they were able to stand up from the chair and return to the initial position was recorded as their test score [30].

The Up-and-Go test measured the time it took for a person to stand up from a standard chair with a seat height of 45 cm (leaning on the back of the chair, with their feet placed flat on the floor behind a marked line), walk three meters at a maximum safe speed, return to the chair, and sit down again. We recorded the time using a stopwatch, and this time was used as the score. The examiner could provide normal walking aids, but no physical assistance was given. The examiner remained close to the participant to prevent falls [31]. This test has an ICC of 0.96 in obese individuals and is strongly correlated with functional mobility.

We used the Short Physical Performance Battery (SPPB) balance test to assess balance [32]. The SPPB test measures balance by asking the participant to stand in three different positions: feet together, one foot slightly wider than the other, and one foot placed in front of the other so that the tip of the first toe is behind the heel of the opposite foot. The test was performed by timing each position for 12 s.

##### Muscle strength

We used the Handgrip test to evaluate muscle strength in participants. The instrument used to measure hand strength was the Jamar dynamometer, and the maximum pressure applied to the dynamometer handle was recorded as hand strength [24]. In this condition, the participant sat on a chair without handles at a suitable height. The tested shoulder was in adduction, without rotation, the elbow was bent at a 90-degree angle, and the forearm was in a neutral position. The wrist was placed between 0 and 20 degrees of extension and 0 to 25 degrees of flexion. The dynamometer handle was set to position number 2, and the maximum pressure applied to the handle was recorded. The average of three attempts was noted as the strength of each hand. To prevent fatigue, rest intervals of 2–3 min were provided between different test phases. For this test, ICC of 0.91–0.98; validated against isokinetic dynamometry.

### Sample size calculation

Since we did not have any previous studies to calculate the effect size for sample size calculation, we used Cohen’s f, one of the most common effect size measures for comparing the means of four groups using one-way analysis of variance. This measure is related to the proportion of variance explained by the group factor (eta-squared). According to Cohen et al. [33], an effect size of f = 0.1 is considered small, f = 0.25 is considered medium, and f = 0.4 is considered large. Therefore, assuming an effect size of f = 0.4 for calculating sample size in a two-group study suggests a large difference between the groups. To achieve statistical significance with a 95% confidence level (α = 0.05) and a statistical power of 80% (β = 20%), it was determined that 19 patients would be required for each group. To account for potential sample loss, a total of 20 patients were included in each group.

After data collection, a post hoc power analysis was conducted using observed effect sizes from our three primary outcomes: insulin, 6-minute walk test (6MWT), and timed up-and-go test. Effect sizes (Cohen’s f) were calculated from partial eta squared (η²) values obtained from the ANOVA. The estimated effect sizes were f = 0.83 for insulin (η² = 0.409), f = 0.62 for 6MWT (η² = 0.276), and f = 0.41 for timed up-and-go (η² = 0.143). Given our total sample size of 80 and α = 0.05, the post hoc power was estimated to be > 0.99 for insulin, > 0.95 for 6MWT, and > 0.90 for timed up-and-go. These results confirm that the study was adequately powered to detect meaningful changes in the primary outcomes.

### Statistical analysis

Data were analyzed using SPSS version 26 and GraphPad Prism for graphical representation. Descriptive statistics (mean ± standard deviation) were calculated for baseline and post-intervention measures. A one-way analysis of variance (ANOVA) was employed to assess between-group differences for continuous variables. When significant differences were found, post-hoc pairwise comparisons using bonferroni test were conducted to identify specific group differences.

To account for potential confounding variables, analysis of covariance (ANCOVA) was applied, adjusting for age as a covariate due to its significant effect on the outcomes (*p* = .007). The assumptions of normality, homogeneity of variances, and homogeneity of regression slopes were verified prior to conducting ANCOVA. Effect sizes (η²) were reported to indicate the magnitude of the differences. Statistical significance was set at *p* < .05 for all analyses.

## Results

All 80 patients participated in the study and completed their follow-up. Table 2 summarizes the baseline characteristics of patients across the four groups. Analysis of variance (ANOVA) indicated no statistically significant difference between the groups in terms of BMI (*p* = .330). However, a significant difference was observed for age (*p* = .007), therefore, age was considered as a covariate in subsequent analyses. Additionally, all participants demonstrated at least 70% adherence to their exercise intervention and there were no compliance and adverse effects reported by the participants after the follow up. Furthermore, all participants exhibited sedentary behavior in terms of their physical activity status before the intervention.

**Table 2:** Comparison of patients’ characteristics between groups (Mean ± SD)

Variables	SLEEVE			BYPASS		*p*-value
	HIIT (*n* = 20)	MICT (*n* = 20)		HIIT (*n* = 20)	MICT (*n* = 20)	
Age (years)	44.35 ± 9.18	46.85 ± 9.36		37.30 ± 10.64	38.20 ± 10.48	0.007
BMI (kg/m2)	42.68 ± 3.30	44.61 ± 3.93		42.65 ± 3.77	44.41 ± 7.14	0.330^NS^

*SD* Standard deviation, *NS *Non-significant, *BMI* Body mass index, *HIIT* High-intensity interval training, *MICT* Moderate-intensity continuous training

Table 3 presents the descriptive statistics of nutritional intake, body composition variables, and cardio metabolic outcomes across the four intervention groups, classified by type of bariatric surgery (sleeve and bypass) and exercise modality (HIIT and MICT). Data are reported as mean (SD) for both baseline and post-intervention assessments. All assumptions required for conducting the analysis of covariance (ANCOVA), including normality, homogeneity of variances, and the homogeneity of regression slopes, were adequately met.

**Table 3:** Descriptive statistics of nutritional intake, body composition factors, cardiometabolic outcomes and physical performance factors by exercise group, Mean (SD)

Variables		SLEEVE			BYPASS			*P*-value*
		HIIT *n* = 20	MICT *n* = 20			HIIT *n* = 20	MICT *n* = 20	
Nutritional Intake	Calorie, Kcal							
	Baseline	2762.95 (390.06)	2653.84 (350.88)			2959.46 (556.45)	3155.87 (962.41)	0.058
	After Intervention	491.32 (221.25)	660.04 (277.89)			588.94 (333.97)	941.84 (43.44)	
	Carbohydrate, gr							
	Baseline	383.67 (85.05)	356.95 (62.99)			399.61 (86.64)	428.75 (179.43)	0.044
	After Intervention	59.06 (34.93)	79.63 (47.06)			66.95 (50.98)	157.26 (17.29)	
	Protein, gr							
	Baseline	93.60 (15.18)	93.72 (18.31)			101.87 (22.54)	99.86 (34.55)	0.595
	After Intervention	24.14 (9.72)	29.80 (13.11)			23.52 (10.59)	27.68 (4.34)	
	Fat, gr							
	Baseline	111.68 (26.86)	104.05 (21.18)			119.85 (38.82)	140.35 (51.42)	0.016
	After Intervention	18.57 (11.11)	25.86 (12.93)			26.36 (18.77)	25.24 (5.75)	
Body Composition Factors	Weight, kg							
	Baseline	108.21 (9.12)	113.96 (15.36)			110.55 (13.98)	116.40 (18.11)	0.299
	After Intervention	91.36 (6.88)	97.31 (11.45)			91.23 (9.63)	96.77 (11.87)	
	BMI, kg/m^2^							
	Baseline	42.68 (3.30)	44.61 (3.93)			42.65 (3.77)	44.71 (7.14)	0.330
	After Intervention	36.03 (2.36)	38.14 (3.05)			35.22 (2.37)	37.15 (4.57)	
	FM, kg							
	Baseline	54.01 (6.66)	56.10 (6.64)			55.06 (9.01)	58.30 (10.13)	0.016
	After Intervention	38.90 (4.82)	42.23 (4.41)			37.97 (5.75)	40.29 (6.00)	
	FFM, kg							
	Baseline	54.24 (4.54)	57.87 (11.81)			55.50 (7.26)	58.10 (11.25)	0.483
	After Intervention	52.46 (4.55)	55.09 (11.62)			53.26 (8.59)	56.48 (11.24)	
	SMM, kg							
	Baseline	19.66 (1.43)	20.06 (1.46)			20.15 (1.53)	20.17 (1.13)	0.630
	After Intervention	18.62 (1.43)	18.75 (1.21)			18.97 (1.50)	18.88 (1.14)	
Cardiometabolic Outcomes	Lipid Profile							
	TG, mg/dL							
	Baseline	146.40 (61.21)	162.60 (123.30)			165.75 (63.83)	166.65 (74.08)	0.860
	After Intervention	125.65 (46.40)	130.45 (68.00)			124.30 (38.25)	133.25 (45.58)	
	Cholesterol, mg/dL							
	Baseline	189.10 (46.25)	191.35 (37.44)			196.55 (31.97)	189.20 (36.37)	0.919
	After Intervention	175.10 (26.93)	188.00 (26.02)			174.35 (19.89)	176.95 (26.15)	
	LDL, mg/dL							
	Baseline	111.55 (27.50)	110.23 (27.65)			109.00 (27.57)	107.40 (30.05)	0.971
	After Intervention	102.95 (14.02)	102.25 (20.04)			91.60 (12.50)	94.25 (16.02)	
	HDL, mg/dL							
	Baseline	52.30 (11.15)	51.20 (11.00)			50.20 (12.48)	47.50 (9.19)	0.558
	After Intervention	54.00 (6.66)	51.80 (8.09)			55.60 (8.67)	49.20 (7.02)	
	Glycemic Indicators							
	FBS, mg/dL	110.60 (15.22)	110.50 (15.67)			112.45 (17.64)	106.65 (16.75)	0.722
	Baseline	95.95 (10.08)	92.90 (9.97)			90.30 (9.09)	93.45 (11.27)	
	After Intervention							
	HbA1C, %							
	Baseline	5.48 (0.62)	5.71 (0.85)			5.57 (1.10)	5.29 (0.78)	0.473
	After Intervention	5.23 (0.56)	5.60 (0.74)			4.97 (0.76)	5.07 (0.65)	
	Insulin, uIU/mL							
	Baseline	21.64 (8.81)	26.07 (5.17)			27.59 (4.59)	28.00 (3.60)	0.004
	After Intervention	14.65 (5.38)	20.45 (3.41)			14.62 (3.25)	17.78 (3.64)	
	Inflammatory Factors							
	hs-CRP, mg/L							
	Baseline	5.10 (2.17)	5.45 (2.04)			6.35 (1.04)	6.05 (1.64)	0.117
	After Intervention	5.05 (1.79)	5.65 (1.60)			4.45 (0.94)	5.35 (1.14)	
	Ferritin, ng/ml							
	Baseline	73.75 (64.07)	85.55 (72.77)			44.00 (37.74)	66.70 (52.66)	0.154
	After Intervention	54.60 (35.65)	58.90 (37.80)			40.80 (24.62)	51.65 (26.89)	
physical performance factors	Aerobic Capacity							
	Walking speed, m/s							
	Baseline	0.93 (0.06)	0.87 (0.05)			0.89 (0.06)	0.88 (0.04)	0.006
	After Intervention	1.07 (0.15)	0.93 (0.04)			0.94 (0.07)	0.91 (0.05)	
	6MWT, meter							
	Baseline	378.10 (25.13)	379.80 (12.53)			384.20 (18.15)	377.05 (16.22)	0.633
	After Intervention	416.15 (27.90)	402.65 (21.52)			406.15 (26.29)	379.05 (18.92)	
	Physical Ability							
	Sit-Stand number, time	3.10 (0.97)	2.80 (0.83)			2.60 (0.94)	2.90 (0.79)	0.352
	Baseline	5.50 (1.05)	5.30 (0.98)			5.05 (1.76)	4.10 (1.74)	
	After Intervention							
	Up-and-Go time, score							
	Baseline	8.70 (0.73)	8.75 (0.64)			8.65 (0.67)	8.35 (0.59)	0.255
	After Intervention	9.40 (0.50)	9.85 (0.75)			10.05 (0.39)	8.90 (1.02)	
	Balance, score							
	Baseline	8.55 (1.05)	8.45 (0.89)			8.55 (1.10)	8.30 (0.73)	0.820
	After Intervention	10.65 (0.99)	9.55 (0.89)			9.10 (1.17)	8.80 (0.89)	
	Muscle Strength							
	Handgrip, Kg							
	Baseline	17.02 (1.46)	17.11 (1.02)			16.98 (1.58)	16.54 (1.06)	0.519
	After Intervention	17.97 (1.69)	18.38 (1.16)			17.96 (1.39)	17.29 (1.05)	

*SD* Standard deviation, *NS* Non-significant, *HIIT* High-intensity interval training, *MICT* Moderate-intensity continuous training, *BMI* Body mass index, *FM* Fat mass, *FFM* Fat free mass, *SMM* Skeletal muscle mass, *FBS *Fasting blood sugar, *HbA1C *Hemoglobin A1C, *LDL* Low-density lipoprotein cholesterol, *HDL* High-density lipoprotein cholesterol, *TG* Triglyceride, *hs-CRP* High-sensitivity quantitative C-reactive protein, *WS *walking speed, *MWT 6* Minutes walking test, *UPGO*Up-and-go test, *BS *Balance test.

* The p-value from ANOVA was used to assess statistical significance

### Food intake

**Fig. 2:**
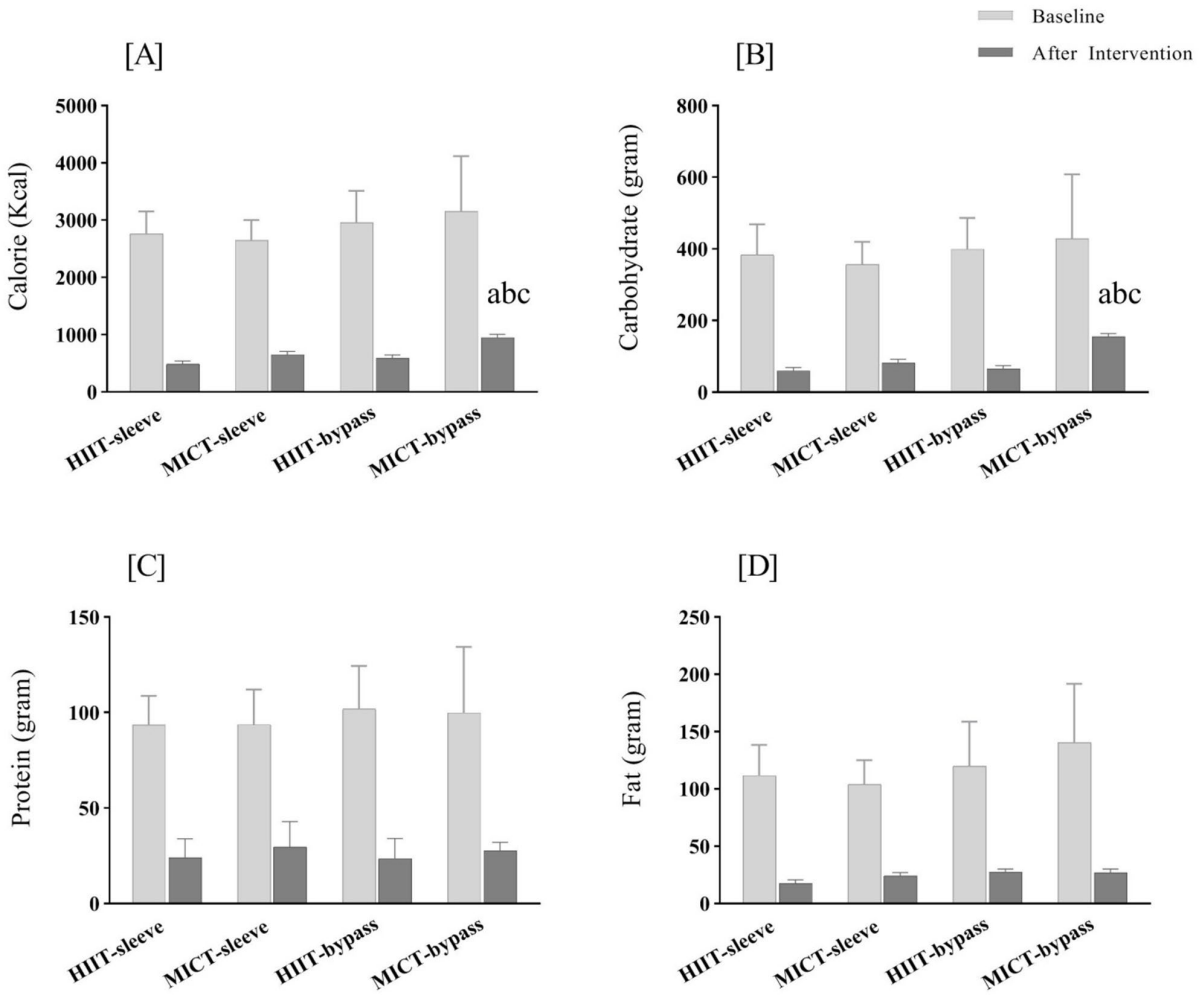
Results of Bonferroni post-hoc test for food intake, **A** calorie (Kcal), **B** Carbohydrate (gram), **C** protein (gram), **D** fat (gram). HIIT *High-Intensity Interval Training*, MICT *Moderate-Intensity Continuous Training. *^a^*p≤ .*05 significant difference with HIIT-sleeve. ^b^ *p*≤ .05 significant difference with MICT-sleeve. ^c^ *p*≤ .05 significant difference with HIIT-bypass. ^d^ *p*≤ .05 significant difference with MICT-bypass

Based on the results of the covariance analysis for dietary intake variables, there was a significant difference mean caloric intake across groups (F_3, 74_= 11.97, *p*< .001, η²= 0.33). Post-hoc comparisons, illustrated in Fig. 2A, revealed that caloric intake in the HIIT-sleeve group was significantly lower than in the MICT-bypass group (*p*< .*001*,* % of change = 95.07%*). Additionally, the MICT-sleeve group exhibited significantly lower caloric intake compared with the MICT-bypass group (*p= .005*,* % of change = 45.74%*). Finally, caloric intake in the HIIT-bypass group was significantly lower than in the MICT-bypass group (*p*< .*001*,* % of change = 60.37%*).

The reported percentages represent between-group differences in adjusted post-intervention means derived from ANCOVA, not within-group changes from baseline. ANCOVA revealed statistically significant between-group differences in carbohydrates intake (F_3, 74_= 23.01, *p*< .001, η²= 0.48). Post-hoc analyses indicated that the HIIT-sleeve interventions significantly greater reduction in carbohydrates compared to MICT-bypass group (*p*< *.001, % of change = 61.1%*). Furthermore, the MICT-sleeve group demonstrated significantly lower carbohydrates intake than the MICT-bypass group (*p*<* .001, % of change = 46.73%*). Similarly, the HIIT-bypass group showed significantly lower carbohydrate intake compared with the MICT-bypass group (*p*< .*001, % of change = 53.93%*) Fig. 2B.

However, direct comparison between groups in protein levels revealed no statistically significant difference (F_3, 74_= 0.60, *p*= .196, η² = 0.06) Fig. 2C. Similarly, no statistically significant difference was observed between groups in fat levels (F_3, 74_= 23.01, *p*< .095, η²= 0.08) Fig. 2D.

### Body composition factors

**Fig. 3:**
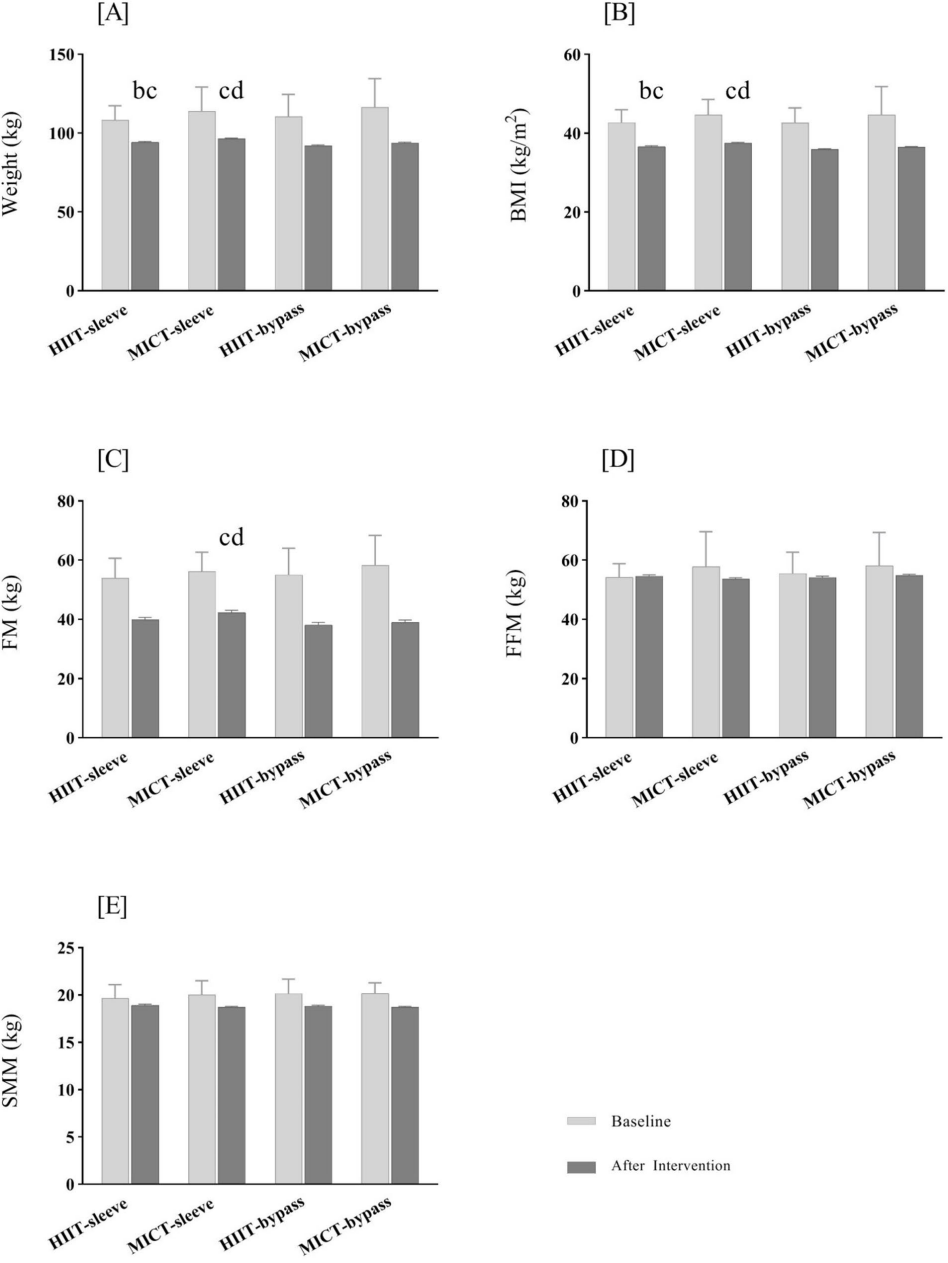
Results of Bonferroni post-hoc test for body composition, **A** weight (kg), **B** body mass index (kg/m2), **C** fat mass (kg), **D** fat free mass (kg), **E** skeletal muscle mass (kg). HIIT *High-Intensity Interval Training*, MICT *Moderate-Intensity Continuous Training. *^a^*p*≤ .05 significant difference with HIIT-sleeve. ^b^*p*≤ .05 significant difference with MICT-sleeve. ^c^*p*≤ .05 significant difference with HIIT-bypass. ^d^*p*≤ .05 significant difference with MICT-bypass

The effects of HIIT and MICT on body composition parameters are detailed in Table 3. Significant differences were observed across the four groups in weight (F_3, 74_= 12.33, *p*< .001, η²= 0.33), BMI (F_3, 74_= 11.95, *p*< .001, η²= 0.33), and FM (F_3, 74_= 4.43, *p*= .006, η²= 0.15), indicating that both the type of exercise and the surgical intervention had an impact. However, no significant differences were found in FFM (F_3, 74_= 1.46, *p*= .223, η²= 0.06) and SMM (F_3, 74_= 1.09, *p*= .359, η²= 0.04) Fig. 3D-E.

Post-hoc analyses indicated that the MICT-sleeve interventions significantly increase in weight compared to HIIT-sleeve (*p*= *.010, % of change = 2.34%*), HIIT-bypass (*p*< *.001*,* % of change = 4.68%*) and MICT-bypass group (*p*= .002,* % of change = 2.85%*). But, HIIT-bypass interventions significantly decrease in weight compared to HIIT-sleeve (*p*= *.018, % of change = 2.23%*). Similarly, MICT-bypass interventions significantly decrease in weight compared to MICT-sleeve (*p*= *.002, % of change = 2.77%*) Fig. 3A. Post-hoc analyses showed that MICT-sleeve significantly increased BMI compared to HIIT-sleeve (*p* = .010), HIIT-bypass (*p* < .001), and MICT-bypass (*p* = .001). In contrast, HIIT-bypass and MICT-bypass significantly decreased BMI compared to their sleeve counterparts (*p* < .001 and *p* = .001), respectively Fig. 3B.

The MICT-sleeve group demonstrated a significantly greater increase in FM compared to the HIIT-bypass (*p*= *.005, % of change = 10.6%*) and MICT-bypass groups (*p*= .037,* % of change = 8.37%*) Fig. 3C.

### Cardiometabolic markers

The effects of HIIT and MICT on serum indicators are detailed in Table 3. Significant differences were observed across the four groups in Cholesterol (F_3, 74_= 4.60, *p*= .005, η²= 0.16), LDL (F_3, 74_= 13.07, *p*< .001, η²= 0.35), HDL (F_3, 74_= 6.97, *p*< .001, η²= 0.22), fasting blood sugar (F_3, 74_= 4.54, *p*= .006, η²= 0.16), HBA1C (F_3, 74_= 18.69, *p*< .001, η²= 0.43), insulin fasting (F_3, 74_= 19.94, *p*< .001, η²= 0.45), hs- CRP (F_3, 74_= 20.49, *p*< .001, η²= 0.45), suggesting that both the type of exercise and the surgical intervention had a significant impact. In contrast, no significant differences were observed in TG (F_3, 74_= 2.04, *p*= .115, η²= 0.08) and ferritin (F_3, 74_= 1.12, *p*= .348, η²= 0.04).

**Fig. 4:**
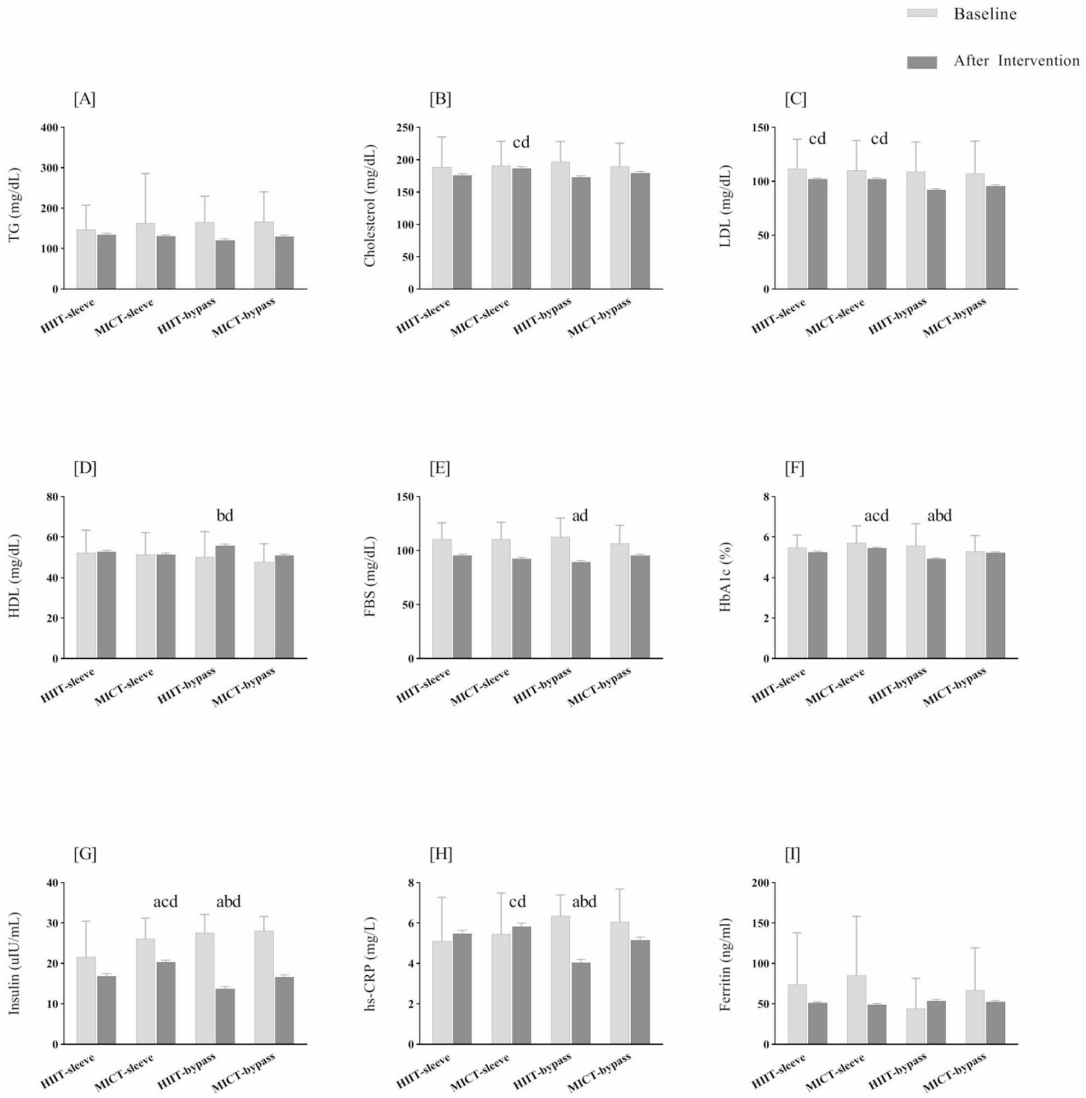
Results of Bonferroni post-hoc test for Serum Indicators, **A** triglyceride (mg/dL), **B** Cholesterol (mg/dL), **C** low density lipoprotein (mg/dL), **D** high density lipoprotein (mg/dL), **E** Fast Blood Sugar (mg/dL), **F** glycated hemoglobin (%), **G** Insulin (uIU/mL), **H** Hs-CRP (mg/L), [I]: Ferritin (ng/ml). HIIT *High-Intensity Interval Training*, MICT *Moderate-Intensity Continuous Training. *^a^*p*≤ .05 significant difference with HIIT-sleeve. ^b^ *p*≤ .05 significant difference with MICT-sleeve. ^c^ *p*≤ .05 significant difference with HIIT-bypass. ^d^ *p*≤ .05 significant difference with MICT-bypass

Post-hoc comparisons, illustrated in Fig. 4B, revealed that cholesterol levels in the MICT-sleeve group were significantly higher than those in the HIIT-bypass (*p*= .034,* % of change = 6.23%*) and MICT-bypass (*p*= .006,* % of change = 8.1%*).

Post-hoc analyses indicated that the HIIT-bypass interventions significantly greater reduction in LDL compared to HIIT-sleeve (*p*< *.001, % of change = 9.77%*) and MICT- sleeve groups (*p*< *.001, % of change = 9.75%*). Furthermore, the MICT-bypass group demonstrated significantly lower LDL than the HIIT-sleeve (*p*= .005,* % of change = 6.35%*) and MICT-sleeve groups (*p*< *.001, % of change = 6.33%*) Fig. 4C. Post-hoc analyses indicated that the HIIT-bypass interventions significantly greater increase in HDL compared to MICT-sleeve (*p*= *.003*,* % of change = 8.48%*) and MICT-bypass groups (*p*= .*001, % of change = 9.29%*) Fig. 4D.

Post-hoc analyses indicated that the HIIT-bypass interventions significantly greater reduction in fasting blood sugar compared to HIIT-sleeve (*p*=* .017*,* % of change = 6.38%*) and MICT- bypass groups (*p*= *.016*,* % of change = 6.30%*) Fig. 4E.

The HIIT-bypass group exhibited significantly lower HBA1C levels compared to the HIIT-sleeve (*p*<* .001, % of change = 6.19%*), MICT-sleeve (*p*< *.001*,* % of change = 9.62%*) and the MICT-bypass groups (*p*< *.001*,* % of change = 5.81%*). Similarly, MICT-bypass significantly lower HBA1C levels compared to the MICT-sleeve (*p*= *.018, % of change = 4.04%*) Fig. 4F.

Post-hoc analyses revealed that the HIIT-bypass intervention led to a significantly greater reduction in insulin fasting compared to the HIIT-sleeve (*p* = .005, % of change = 18.66%), MICT-sleeve (*p* < .001, % of change = 32.37%) and MICT- bypass groups sleeve (*p* = .003, % of change = 17.59%). Insulin fasting was significantly reduced in the HIIT-sleeve group compared to the MICT-sleeve group (*p* = .001, % of change = 16.85%). Furthermore, Insulin fasting was significantly reduced in the MICT-bypass group compared to the MICT-sleeve group (*p* < .001, % of change = 17.93%) Fig. 4G.

Finally, HIIT-bypass interventions significantly greater reduction in hs-CRP compared to HIIT-sleeve (*p*< .001,* % of change = 26.20%*) and MICT-sleeve (*p*< *.001*,* % of change = 30.63%*) and MICT-bypass groups (*p*< .*001*,* % of change = 21.43%*). Furthermore, MICT-bypass interventions significantly greater reduction in hs-CRP compared to MICT-sleeve (*p*= .030,* % of change = 11.70%*) Fig. 4H.

It is worth noting that all cardiometabolic markers that have significantly changed, have met their Minimal Clinically Important Difference (MCID) value, except for FBS, which the MCID range is between 7 and 20%.

### Physical performance

The impacts of HIIT and MICT on physical performance including aerobic capacity, physical ability and muscle strength are detailed in Table 3.

#### Aerobic capacity

**Fig. 5:**
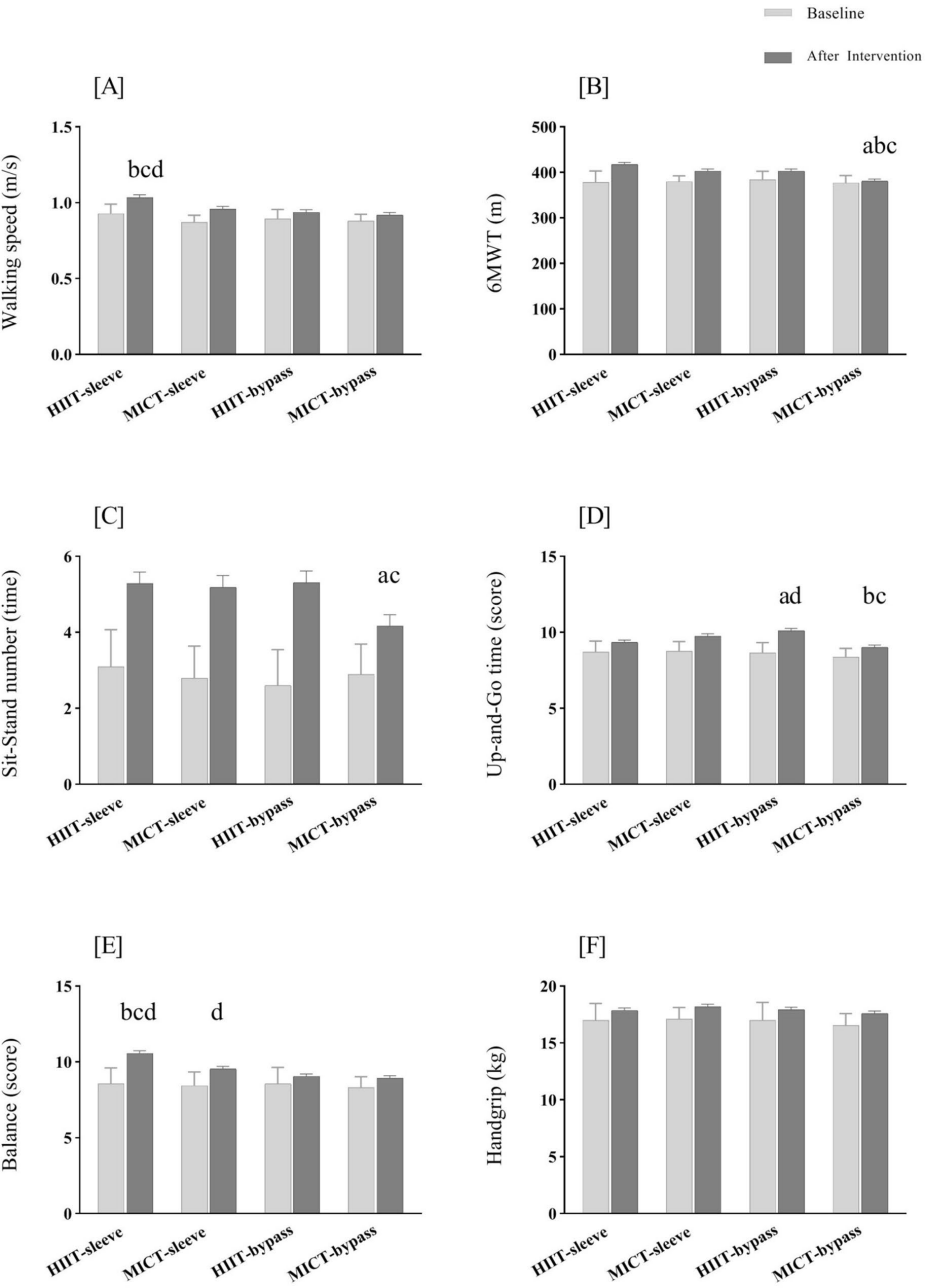
Results of Bonferroni post-hoc test for Physical Performance, **A** Walking speed (m/s), **B** 6MWT (meter), **C** Sit-Stand number (time), **D** Up-and-Go time (score), **E** balance (score), **F** hand grip (kg). HIIT *High-Intensity Interval Training*, MICT *Moderate-Intensity Continuous Training. *^a^ *p*≤ .05 significant difference with HIIT-sleeve. ^b^ *p*≤ .05 significant difference with MICT-sleeve. ^c^ *p*≤ .05 significant difference with HIIT-bypass. ^d^ *p*≤ .05 significant difference with MICT-bypass.

Significant differences were observed across the four groups in walking speed (F_3, 74_= 8.70, *p*< .001, η²= 0.26). The HIIT-sleeve group demonstrated a significant improvement in walking speed compared to the MICT-sleeve (*p*= *.012, % of change = 8.04%*), HIIT-bypass (*p*= .001,* % of change = 10.34%*) and MICT-bypass groups (*p*< *.001, % of change = 12.62%*) Fig. 5A.

Significant differences were observed across the four groups in 6MWT (F_3, 74_= 10.47, *p*< .001, η²= 0.30). The HIIT-sleeve group demonstrated a significant improvement in 6MWT compared to the MICT-bypass groups (*p*< *.001, % of change = 9.63%*). Furthermore, MICT-sleeve group demonstrated a significant improvement in 6MWT compared to the MICT-bypass groups (*p*= .*010, % of change = 5.81%*) Fig. 5B.

#### Physical ability

Significant differences were observed across the four groups in sit-to-stand test (F_3, 74_= 3.35, *p*= .023, η²= 0.12). The HIIT-bypass group demonstrated a significant improvement in sit-to-stand test compared to the MICT-bypass groups (*p*= .041,* % of change = 27.47%*), in addition to HIIT-sleeve greater improvement over MICT-bypass (*p*=.049, * % of change = 26.97%*) Fig. 5C.

A significant group effect was observed for the timed up-and-go test (F_3, 74_= 9.53, *p*< .001, η²= 0.28). Post-hoc comparisons revealed that the HIIT-bypass group exhibited a significantly greater improvement in timed up-and-go performance compared with the HIIT-sleeve (*p*= .006,* % of change = 8.21%*) and MICT-bypass groups (*p*< *.001, % of change = 12.21%*). Similarly, MICT-sleeve significantly greater improvement in timed up-and-go performance compared with the MICT-bypass (*p*= .013,* % of change = 8.16%*) Fig. 5D. Finally, Significant differences were observed across the four groups in balance (F_3, 74_= 20.38, *p*< .001, η²= 0.46). Post-hoc analyses revealed that the HIIT-sleeve intervention led to a significant improvement in balance compared to the MICT-sleeve (*p*< .001, % of change = 10.85%), HIIT-bypass sleeve (*p* = .003, % of change = 16.83%), MICT- bypass groups sleeve (*p*< *.001, *% of change = 18.34%). Furthermore, MICT-sleeve group demonstrated a significant improvement in balance compared to the MICT-bypass groups (*p*= .*047, % of change = 6.76%*) Fig. 5E.

#### Muscle strength

Our results showed no statistically significant difference was observed between groups in handgrip test (F_3, 74_= 1.34, *p*< .267, η²= 0.05) Fig. 5F.

It is worth noting that all physical performance factors that have significantly changed, have met their MCID value, except for timed up-and-go test, which the MCID range is between 15 and 25%.

## Discussion

This study aimed to investigate the comparison impact of 8 weeks of HIIT and MICT protocols on body composition parameters, cardiometabolic indicators, and physical performance in patients with sarcopenic obesity undergoing bariatric surgery. After adjusting for age, the current study revealed higher preference for HIIT, with more favorable impact on certain physical and metabolic outcomes, including aerobic capacity (walking speed), physical ability (balance, timed up-and-go, and sit-to stand tests) glycemic indicators (FBS, insulin, HbA1c), and some inflammation and lipid parameters (hs-CRP and HDL) compared to the MICT protocol. Additionally, women with HIIT training had significant reduction in their weight and BMI compared to those who followed MICT training protocol. A larger sample size and matching surgery types between the two training groups can further strengthen the study. No compliance issues or adverse effects were reported by participants during follow-up for either the HIIT or MICT training protocols. This further emphasizes that HIIT might be a feasible and safe approach for supervised sarcopenic patients with BS, offering logistical advantages, such as shorter training durations compared to MICT. However, because the two primary factors influencing the management of sarcopenic obesity, SSM and muscle strength, did not differ significantly between the two training modalities, and because the apparent superiority of HIIT in some factors was dependent on the type of surgery, caution should be exercised when interpreting our findings regarding the superior effects of HIIT over MICT. Further studies are needed to provide more conclusive evidence.

As far as we know, no studies have directly investigated the effects of HIIT and MICT, or compared them, in post-bariatric patients with sarcopenic obesity. However, there are conflicting results regarding the impact of HIIT and MICT on body composition measurements and physical performance in other populations [19], as well as in animal models [17, 18], which most studies with longer-term interventions showing more significant changes. For example, two studies in rats with sarcopenic obesity revealed no changes in body composition after 4 [18] and 8 [17] weeks of HIIT and MICT interventions, but showed significant differences in the regulation of muscle-related gene expression. In the former study [18], HIIT led to greater myocardial protection; in the latter [17], MICT caused slower protein breakdown. On the other hand, a study in older adults with obesity [19], which might resemble sarcopenic obesity, reported greater improvements in some physical performance factors along with body composition changes, with HIIT providing greater benefits in aerobic capacity (6MWT and walking speed), physical ability (chair and step tests), lean mass preservation, and skeletal muscle mitochondrial adaptation, while MICT was superior in lower-limb strength and fat mass reduction. Although previous studies showed mix results for MICT and HIIT effect, the result of current study revealed that after 8 weeks of intervention, there was a significant improvement in aerobic capacity, physical ability tests, weight, and BIM reduction for HIIT than MICT, but no significant changes in muscle strength and body composition factors, especially SMM, in both trainings. However, our patient with SO showed more FM reduction in HIIT-bypass group over the others, but this effect was not statistically significant.

Additionally, two reviews on sarcopenia alone [34, 35] have illustrated that HIIT plays a beneficial role in enhancing physical ability, aerobic capacity, and muscle strength compared to other forms of training, such as MICT and continuous aerobic exercise. These align with the findings of the current study, except for muscle strength, which didn’t change with both types of training.

Overall, our study supports the previous consistent results regarding the positive effect of HIIT over MICT in improving physical ability and aerobic capacity, but, due to mixed results regarding the effectiveness of HIIT and MICT on muscle strength, further studies are needed to clarify the lack of change observed in our study. Similarly, there are inconsistent results regarding the effects of HIIT and MICT on FM, FFM, and SMM both after short-term or long-term interventions [19, 36, 37]. The exact reason for these heterogeneous findings is unclear but could be related to differences in sample size, various biological responses in animal and human models, beside the duration of the intervention. Notably, our findings after 8 weeks of following the established post-bariatric rehabilitation guidelines, which recommend a minimum of 150 min per week of moderate-to-vigorous physical activity, revealed a 5% greater reduction with HIIT than with MICT. The reduction in weight and BMI can be important for weight loss outcomes after bariatric surgery, even though it was not accompanied by changes in FM, FFM, SMM, as an effective factors for SO management, in either training group.

The mechanisms underlying the effects of different exercise types on physical function remain incompletely understood. However, studies have shown that various exercise modalities can positively impact physical capacity and muscular function in patients with sarcopenic obesity, not only through FM reduction but also through muscle fiber remodeling at two levels: (1) quantity, involving reduced skeletal muscle atrophy and increased muscle mass; and (2) quality, encompassing morphological changes such as alterations in muscle size, mitochondrial content, and neuromuscular capacity [16, 19]. In the current study, physical performance improvements were not accompanied by differences in FFM and SMM between the two training protocols. Thus, we hypothesize that probably after the 8-week intervention, our patients with sarcopenic obesity undergone HIIT experienced slightly greater improvements in muscle quality than quantity, contributing to enhanced physical performance (aerobic capacity and physical ability), compared to MICT, even without changes in body composition. Previous studies have reported that HIIT positively affects morphological changes, including mitochondrial biogenesis and neuromuscular capacity (e.g., increased muscle activation and isometric quadriceps strength) in sarcopenic patients [16, 38]. However, as handgrip tests in our patients showed no significant differences between the two training protocols, uncertainty persists regarding HIIT’s superiority over MICT for improving overall physical performance in this population. Notably, since we did not collect adipose or muscle tissue biopsies to assess gene expression, mitochondrial content, or other quality changes in these tissues, the underlying mechanisms of these findings remain unclear. Further studies, particularly in sarcopenic obesity, are needed.

Regarding cardiometabolic factors, our findings support previous studies: one is a meta-analysis in various clinical populations (analyzing 7 randomized trials involving 182 participants, mostly 12–16 weeks) and the other in obese elderly women (16 weeks of intervention) [39, 40]. These two studies indicated that HIIT is more effective than MICT in enhancing certain cardiometabolic parameters, including, insulin resistance, FBS, HbA1C, hs-CRP, and lipid profiles. In the current study, we observed a significant improvement in all glycemic indicators (insulin level, FBS, HbA1c) and several inflammatory (hs-CRP) and lipid profiles (HDL) in women with SO who followed HIIT compared to MICT after 8 weeks of intervention. Additionally, our patients with SO showed greater LDL and cholesterol reduction in the HIIT-bypass group over the others, but this effect was not statistically significant. On the other hand, the current findings did not support the results of the Youssef L et al. study [19] in older adults with obesity, which reported that TG is the only blood factor related to metabolic syndrome that decreased by 8.8% in HIIT compared to MICT after 12 weeks of intervention. We only observed HDL changes in the lipid profile along with glycemic and inflammatory factors between the two types of training.

The significant enhancement in cardiovascular parameters in the HIIT supports the suggested mechanisms that HIIT (vs. MICT) may enhance vascular and glycemic metabolites by increasing GLUT4 translocation and improving insulin sensitivity, leading to greater glucose uptake and potentially improved insulin levels [20, 39]. Although insulin sensitivity was not directly assessed in this study, the measured insulin levels in our study were significantly reduced by a mean of 24.98% in the HIIT group (especially in those with bypass) compared to the MICT group, further confirming the proposed mechanisms. Additionally, HIIT may promote nitric oxide (NO) bioavailability, resulting in reduced inflammation levels [41]. Although, as noted in a meta-analysis by Ramos et al. [39], longer exercise intervention periods may be required to induce significant changes in vascular-related factors, the 8 weeks of intervention in the current study nevertheless provided cardiovascular factors improvements in our participants.

It is important to note that, when comparing the two types of surgery across both training protocols, greater enhancements in the sit-to-stand test, weight, BMI, and food intake were observed in the HIIT groups (for both surgery types) compared to MICT. Conversely, greater reductions in cardiometabolic indicators and the timed up-and-go test were observed in the HIIT-bypass surgery group, while improvements in walking speed and balance were more significant in the HIIT-sleeve group than in the others. It remains unclear why certain improvements were observed between the HIIT and MICT groups in the bypass surgery but not in the sleeve group, and vice versa. Although in the current study, the surgery type was matched between two types of training, further studies are needed to clarify this discrepancy.

The study has several limitations. First, it is a prospective quasi-experimental study with no control group, so a well-designed clinical trial is needed. Second, this study included only one sex (female), which limits the generalizability of the conclusions for all BS candidates. Third, the intervention period was limited (8 weeks), which may not have allowed for sufficient influence on some serum parameters and may restrict conclusions about long-term outcomes. Fourth, some serum profiles, such as inflammatory factors, were incomplete, as we were only able to assess hs-CRP and ferritin levels. Fifth, due to financial limitations, only some factors for inclusion and exclusion criteria were assessed. Although important contributory factors were considered (such as age), more comprehensive factors for inclusion and exclusion criteria as well as baseline characteristics such as comorbidities and sarcopenia severity are needed. Sixth, a blinded study would be beneficial for future research. Seventh, we suggest that subsequent studies evaluate changes in body composition factors and assess molecular biomarkers of obesity-related sarcopenia. Eighth, BIA is not the definitive standard for measuring body composition, but it is a highly non-invasive, cost-effective, safe, and reliable method. Additionally, studies comparing BIA to DXA, the gold standard, have demonstrated a strong correlation between the two for assessing human body composition [42]. Ninth, small effect sizes in certain outcomes may suggest variability in response to intervention and limited statistical power for those variables, warranting cautious interpretation and further research. Lastly, potential self-report bias in dietary assessments and the lack of objective verification, such as heart rate monitor data for home exercises beyond logbook records, should also be considered.

This study has also several strengths. First, it benefits from a large sample size with comparing the effects of two exercise protocols in patients undergoing two common types of BS procedures and matches the participants based on sleeve and bypass surgery. Second, it is one of the few human-based evidence studies to determine the optimal physical activity program, between HIIT and MICT, for patients with sarcopenic obesity, especially after BS.

## Conclusion

Based on the current study, both training protocols (HIIT and MICT) are viable options for post-bariatric patients with sarcopenic obesity, offering valuable and safe options for public health program implementation. Our findings indicate that after adjusting for age, HIIT emerges as a more beneficial rehabilitation strategy, enhancing aerobic capacity (walking speed test), physical ability (up-and-go, sit-to-stand, and balance tests), cardiometabolic markers (glycemic indicators, hs-CRP, and HDL), as well as reducing weight and BMI, and calorie and carbohydrate intakes, compared to the MICT protocol. The benefits of HIIT, achieved in shorter exercise sessions, may offer advantages for program design and patient adherence. Program administrators should consider patient-specific factors, as well as the surgery type, when choosing between HIIT and MICT approaches. However, because there were no differences between MICT and HIIT in muscle strength and body composition outcomes (FFM and SMM), further studies are needed in this area. Future research should explore the long-term effects of these interventions in well-designed clinical trials and should address the underlying molecular mechanisms.

## Data Availability

The datasets used and/or analyzed during the current study are available from the corresponding author on reasonable request.

## Abbreviations

BS: Bariatric surgery
HIIT: High intensity interval training
MICT: Moderate-intensity continuous training
FM: Fat Mass
SMM: Skeletal muscle mass
FFM: Fat free mass
6MWT: 6-minute walking test
HDL-c: high-density lipoprotein cholesterol
LDL-c: Low-density lipoprotein cholesterol
SPPB: Short Physical Performance Battery
hs-CRP: High-sensitivity quantitative C-reactive protein
HbA1c: Glycated hemoglobin
FBS: Fasting blood sugar
ANOVA: One way analysis of variance
ASM: Appendicular Skeletal Muscle mass
GPAQ: Global physical activity questionnaire
ACSM: American college of sports medicine

## References

[1] Bužga M, Marešová P, Petřeková K, Holéczy P, Kuča K. The efficacy of selected bariatric surgery methods on lipid and glucose metabolism: a retrospective 12-month study. Cent Eur J Public Health. 2018;26(1):49–53. 29684298 10.21101/cejph.a4637

[2] Bužga M, Švagera Z, Tomášková H, Hauptman K, Holéczy P. Metabolic effects of sleeve gastrectomy and laparoscopic greater curvature plication: an 18-month prospective, observational, open-label study. Obes Surg. 2017;27:3258–66. 28674838 10.1007/s11695-017-2779-2

[3] Ciangura C, Bouillot JL, Lloret-Linares C, Poitou C, Veyrie N, Basdevant A, et al. Dynamics of change in total and regional body composition after gastric bypass in obese patients. Obesity. 2010;18(4):760–5. 19834464 10.1038/oby.2009.348

[4] Nuijten MA, Monpellier VM, Eijsvogels TM, Janssen IM, Hazebroek EJ, Hopman MT. Rate and determinants of excessive fat-free mass loss after bariatric surgery. Obes Surg. 2020;30:3119–26. 32415634 10.1007/s11695-020-04654-6 PMC7305251

[5] Mousavi M, Tabesh MR, Khalaj A, Eini-Zinab H, Jahromi SR, Abolhasani M. Food addiction disorder 2 years after sleeve gastrectomy; association with physical activity, body composition, and weight loss outcomes. Obes Surg. 2021;31:3444–52. 33934295 10.1007/s11695-021-05420-y

[6] Mastino D, Robert M, Betry C, Laville M, Gouillat C, Disse E. Bariatric surgery outcomes in sarcopenic obesity. Obes Surg. 2016;26:2355–62. 26926186 10.1007/s11695-016-2102-7

[7] Johnson Stoklossa CA, Sharma AM, Forhan M, Siervo M, Padwal RS, Prado CM. Prevalence of sarcopenic obesity in adults with class II/III obesity using different diagnostic criteria. J Nutr metabolism. 2017;2017(1):7307618. 10.1155/2017/7307618 PMC5380855 28421144

[8] Gagnon C, Schafer AL. Bone health after bariatric surgery. J Bone Mineral Res Plus. 2018;2(3):121–33. 10.1002/jbm4.10048 PMC6124196 30283897

[9] de Holanda NCP, Baad VMA, Bezerra LR, de Lima SKM, Filho JM, de Holanda Limeira CC, et al. Secondary hyperparathyroidism, bone density, and bone turnover after bariatric surgery: differences between Roux-en-Y gastric bypass and sleeve gastrectomy. Obes Surg. 2021;31:5367–75. 34635988 10.1007/s11695-021-05739-6

[10] Assyov Y, Nedeva I, Spassov B, Gerganova A, Velikov T, Kamenov Z, et al. Nutritional management and physical activity in the treatment of sarcopenic obesity: a review of the literature. Nutrients. 2024;16(15):2560. 39125439 10.3390/nu16152560 PMC11314398

[11] Reiter L, Bauer S, Traxler M, Schoufour JD, Weijs PJ, Cruz-Jentoft A, et al. Effects of nutrition and exercise interventions on persons with sarcopenic obesity: an umbrella review of meta-analyses of randomised controlled trials. Curr Obes Rep. 2023;12(3):250–63. 37249818 10.1007/s13679-023-00509-0 PMC10482763

[12] Janani S, Sedhunivas R. Effectiveness of exercise interventions on muscle mass among older adults with sarcopenic obesity: A scoping review. Aging Med. 2024;7(1):115–20. 10.1002/agm2.12288 PMC10985769 38571676

[13] Villa-González E, Barranco-Ruiz Y, Rodríguez-Pérez MA, Carretero-Ruiz A, García-Martínez JM, Hernández-Martínez A, et al. Supervised exercise following bariatric surgery in morbid obese adults: CERT-based exercise study protocol of the EFIBAR randomised controlled trial. BMC Surg. 2019;19:1–12. 31488115 10.1186/s12893-019-0566-9 PMC6729089

[14] Bayles MP. ACSM’s exercise testing and prescription. Lippincott williams & wilkins, Philadelphia, USA. 2023.

[15] Tabesh MR, Eghtesadi M, Abolhasani M, Maleklou F, Ejtehadi F, Alizadeh Z. Nutrition, physical activity, and prescription of supplements in pre-and post-bariatric surgery patients: an updated comprehensive practical guideline. Obes Surg. 2023;33(8):2557–72. 37389806 10.1007/s11695-023-06703-2

[16] Morcillo-Losa JA, Díaz-Martínez MP, Ceylan Hİ, Moreno-Vecino B, Bragazzi NL, Párraga Montilla J. Effects of high-intensity interval training on muscle strength for the prevention and treatment of sarcopenia in older adults: a systematic review of the literature. J Clin Med. 2024;13(5):1299. 38592165 10.3390/jcm13051299 PMC10931549

[17] Hong W, Tian H, Luan Y, Ma Y, Xiong Y, Zhang B. Effects of moderate-intensity continuous training and high-intensity interval training on obesity-related muscle atrophy in mice. Chin J Tissue Eng Res. 2024;28(35):5618.

[18] de Oliveira França G, Frantz EDC, Magliano DAC, Bargut TCL, Sepúlveda-Fragoso V, Silvares RR, et al. Effects of short-term high-intensity interval and continuous exercise training on body composition and cardiac function in obese sarcopenic rats. Life Sci. 2020;256:117920. 32522571 10.1016/j.lfs.2020.117920

[19] Youssef L, Granet J, Marcangeli V, Dulac M, Hajj-Boutros G, Reynaud O, et al. editors. Clinical and biological adaptations in obese older adults following 12-weeks of high-intensity interval training or moderate-intensity continuous training. Healthcare: MDPI; 2022. 10.3390/healthcare10071346 PMC9315493 35885872

[20] de Oliveira França G, Frantz EDC, Bargut TCL, Sepúlveda-Fragoso V, Silvares RR, Daliry A, et al. Effects of short-term high-intensity interval and continuous exercise training on body composition and cardiac function in obese sarcopenic rats. Life Sci. 2020;256:117920. 32522571 10.1016/j.lfs.2020.117920

[21] Idris I, Anyiam O. The latest evidence and guidance in lifestyle and surgical interventions to achieve weight loss in people with overweight or obesity. Diabetes Obes Metabolism. 2025;27:20–34. 10.1111/dom.16296 PMC12000859 40026042

[22] Kim JH, Choi SH, Lim S, Kim KW, Lim JY, Cho NH, et al. Assessment of appendicular skeletal muscle mass by bioimpedance in older community-dwelling Korean adults. Arch Gerontol Geriatr. 2014;58(3):303–7. 24309033 10.1016/j.archger.2013.11.002

[23] Chen L-K, Woo J, Assantachai P, Auyeung T-W, Chou M-Y, Iijima K, et al. Asian Working Group for Sarcopenia: 2019 consensus update on sarcopenia diagnosis and treatment. J Am Med Dir Assoc. 2020;21(3):300–7. e2. 32033882 10.1016/j.jamda.2019.12.012

[24] Ad AF, Natali AJ, Vieira BC, Valle MAANd G, Moreira D, Massy-Westropp N, et al. The relationship between hand grip strength and anthropometric parameters in men. Arch de Med del Deporte. 2014;161(31):160–4.

[25] Organization WH. Global physical activity questionnaire (GPAQ) WHO13 November 2021 Technical document. Available from: https://www.who.int/publications/m/item/global-physical-activity-questionnaire

[26] Mohebi F, Mohajer B, Yoosefi M, Sheidaei A, Zokaei H, Damerchilu B, et al. Physical activity profile of the Iranian population: STEPS survey, 2016. BMC Public Health. 2019;19:1–17. 31519165 10.1186/s12889-019-7592-5 PMC6743153

[27] Moghaddam MB, Aghdam FB, Jafarabadi MA, Allahverdipour H, Nikookheslat SD, Safarpour S. The Iranian version of international physical activity questionnaire (IPAQ) in Iran: content and construct validity, factor structure, internal consistency and stability. World Appl Sci J. 2012;18(8):1073–80.

[28] Castell GS, Serra-Majem L, Ribas-Barba L. What and how much do we eat? 24-hour dietary recall method. Nutr Hosp. 2015;31(3):46–8. 25719770 10.3305/nh.2015.31.sup3.8750

[29] Crispim Carvalho NN, Martins VJB, Filho JM, de Arruda Neta ACP, Pimenta FCF, de Brito Alves JL. Effects of preoperative sarcopenia-related parameters on the musculoskeletal and metabolic outcomes after bariatric surgery: a one-year longitudinal study in females. Sci Rep. 2023;13(1):13373. 37591922 10.1038/s41598-023-40681-w PMC10435473

[30] Bohannon RW. Reference values for the five-repetition sit-to-stand test: a descriptive meta-analysis of data from elders. Percept Mot Skills. 2006;103(1):215–22. 17037663 10.2466/pms.103.1.215-222

[31] Higashi Y, Yamakoshi K, Fujimoto T, Sekine M, Tamura T. Quantitative evaluation of movement using the timed up-and-go test. IEEE Eng Med Biol Mag. 2008;27(4):38–46.18270049

[32] Gómez JF, Curcio C-L, Alvarado B, Zunzunegui MV, Guralnik J. Validity and reliability of the Short Physical Performance Battery (SPPB): a pilot study on mobility in the Colombian Andes. Colombia Med. 2013;44(3):165–71. PMC4002038 24892614

[33] Cohen J. Statistical power analysis for the behavioral sciences. Routledge, London, UK. 2013.

[34] Liu Q-Q, Xie W-Q, Luo Y-X, Li Y-D, Huang W-H, Wu Y-X et al. High intensity interval training: a potential method for treating sarcopenia. Clin Interv Aging. 2023;17:857–72. 10.2147/CIA.S366245 PMC9152764 35656091

[35] Hayes LD, Elliott BT, Yasar Z, Bampouras TM, Sculthorpe NF, Sanal-Hayes NE, et al. High intensity interval training (HIIT) as a potential countermeasure for phenotypic characteristics of sarcopenia: A scoping review. Front Physiol. 2021;12:715044. 34504439 10.3389/fphys.2021.715044 PMC8423251

[36] Martins C, Kazakova I, Ludviksen M, Mehus I, Wisloff U, Kulseng B, et al. High-intensity interval training and isocaloric moderate-intensity continuous training result in similar improvements in body composition and fitness in obese individuals. Int J Sport Nutr Exerc Metab. 2016;26(3):197–204. 26479856 10.1123/ijsnem.2015-0078

[37] Viana RB, Naves JPA, Coswig VS, De Lira CAB, Steele J, Fisher JP et al. Is interval training the magic bullet for fat loss? a systematic review and meta-analysis comparing moderate-intensity continuous training with high-intensity interval training (HIIT). Br J Sports Med. 2019;53:655–64. 10.1136/bjsports-2018-099928 30765340

[38] Zhu Y, Zhou X, Zhu A, Xiong S, Xie J, Bai Z. Advances in exercise to alleviate sarcopenia in older adults by improving mitochondrial dysfunction. Front Physiol. 2023;14:1196426. 37476691 10.3389/fphys.2023.1196426 PMC10355810

[39] Ramos JS, Dalleck LC, Tjonna AE, Beetham KS, Coombes JS. The impact of high-intensity interval training versus moderate-intensity continuous training on vascular function: a systematic review and meta-analysis. Sports Med. 2015;45:679–92. 25771785 10.1007/s40279-015-0321-z

[40] Rohmansyah NA, Ka Praja R, Phanpheng Y, Hiruntrakul A. High-intensity interval training versus moderate-intensity continuous training for improving physical health in elderly women. INQUIRY: J Health Care Organ Provis Financing. 2023;60:00469580231172870. 10.1177/00469580231172870 PMC10184247 37158072

[41] Pirani H, Bakhtiari A, Amiri B, Salehi OR. Beneficial mitochondrial biogenesis in gastrocnemius muscle promoted by high-intensity interval training in elderly female rats. Cell J (Yakhteh). 2022;25(1):11. 10.22074/CELLJ.2022.557565.1078 PMC9868433 36680479

[42] Kim H-S, Kim S-Y. Validity of bioelectrical impedance analysis (BIA) in measurement of human body composition. Clin Experimental Pediatr. 2005;48(7):696–700.

